# Efficacy and safety of warm needle treatment for scapulohumeral periarthritis: A protocol for systematic review and meta-analysis

**DOI:** 10.1097/MD.0000000000023237

**Published:** 2020-11-20

**Authors:** Xiaoyu Wang, Xinghua Hai, Dongli Jiang, Lianjun Yin, Huanan Li, Qi Wang, Fang Liu, Guoqiang Xu, Qing Sun

**Affiliations:** a First Teaching Hospital of Tianjin University of Traditional Chinese Medicine, Tianjin; b Acupuncture and Rehabilitation Clinical College, Guangzhou University of Chinese Medicine; c Recovery Unit, The Third Affiliated Hospital of Southern Medical University, Guangzhou; d Department of Public Health and Preventive Medicine, Baotou Medical College, Baotou.

**Keywords:** meta-analysis, protocol, scapulohumeral periarthritis, systematic review, warm needle acupuncture

## Abstract

**Background:**

To evaluate the effectiveness and safety of warm needle acupuncture (WNA) treatment for Scapulohumeral periarthritis.

**Methods:**

Relevant randomized controlled trials will be searched from the databases of Pubmed, the Cochrane Library, Embase, CNKI, Wanfang Database, CBM and VIP Database from their inception to September 2021. The primary outcomes are effective rate, visual analog scale score. The secondary outcomes are Constant-Murley score, Japanese Orthopaedic Association scores, adverse events. Two reviewers will independently select studies, collect data, and assess the methodology quality by the Cochrane risk of bias tool. The Stata 14.0 will be used for meta-analysis.

**Results:**

This study is ongoing and will be submitted to a peer-reviewed journal for publication.

**Conclusion:**

This study will provide an assessment of the current state of WNA for the scapulohumeral periarthritis, aiming to show the efficacy and safety of WNA treatment.

**Ethics and dissemination:**

There is no requirement of ethical approval and informed consent, and it will be in print or published by electronic copies.

**Registration:**

INPLASY2020100049

## 1 Introduction

### 1.1 Description of the condition

Scapulohumeral periarthritis (SP) is a chronic, sterile, specific inflammatory reaction caused by injury or degeneration of the joint capsule around the shoulder joint and its surrounding ligaments, tendons, bursae, muscles and other soft tissues.^[1–3]^The incidence of SP has been reported to be 2% to 5% in some studies,^[4,5]^ and 5% to 8.79% in China,^[6]^ with a higher incidence in women than in men (about 3:1) and is characterized by slow onset and long duration of disease,^[7]^ with a high incidence at the age of 40 to 70 years old and around 50 years old,^[8]^ with more women than men,^[9]^ bringing tremendous benefits to patients’ daily life and work. The pain, especially at night, affects sleep and can lead to depression or anxiety in the long run.^[10–12]^ Shoulder pain and limitation of movement affects all aspects of a person's life, suggesting that treatment of this condition is essential.

The current treatment of SP is mainly conservative, with non-surgical treatment consisting of oral non-steroidal medications, intra-articular injections of tretinoin, bupivacaine suprascapular nerve blocks.^[13,14]^ Although shoulder pain and joint motion improve in the short term, most of the effects disappear within 4 weeks after treatment.^[15,16]^ Besides, joint cavity injections may result in serious adverse events such as infectious arthritis and cartilage damage.^[17]^

### 1.2 Description of the intervention

Warm Needle Acupuncture (WNA) is an important part of Traditional Chinese Medicine acupuncture therapy in which the acupuncturist adds a warm stimulus, such as a burning moxa stick, to the needle handle to deliver heat and needle sensation to the deeper tissues, thereby enhancing the therapeutic effect.^[18]^ It combines the technical advantages of acupuncture and moxibustion to produce better therapeutic results and is a safe and effective treatment without any toxic side effects.^[19]^ Evidence from relevant studies shows that WNA can effectively relieve chronic pain,^[20]^ such as lumbar disc herniation,^[21]^ rheumatoid arthritis,^[22]^ can strengthen the immune system,^[23]^ improve blood circulation,^[24]^ and help in the treatment and recovery of chronic pain conditions.

To our knowledge, a large number of studies have shown that WNA has a significant effect on the improvement of pain and shoulder function in SP patients.^[25]^ However, the relationship between WNA and SP has not been elucidated. Therefore, our aim in this review was to conduct a systematic review and meta-analysis to assess the effectiveness and safety of WNA for the SP.

## 2 Methods and analysis

### 2.1 Study registration

The protocol has been registered on the International Platform of Registered Systematic Review and Meta-analysis Protocols (INPLASY) registration number, INPLASY2020100049 (https://inplasy.com/inplasy-2020-10-0049/) basing on the Preferred Reporting Items for Systematic Reviews and Meta-Analyses Protocols (PRISMA-P) statement guidelines.^[26]^

### 2.2 Study eligibility criteria

#### 2.2.1 Type of studies

All randomized controlled trials evaluate the effectiveness and safety of WNA in the treatment of SP will be included. There is no uniform requirement for the language of the survey results.

#### 2.2.2 Type of participants

Patients diagnosed with SP are not restricted by age, sex, race, occupation, education, aetiology, and severity etc. The diagnostic criteria must be clear.

#### 2.2.3 Type of interventions

The purpose of the study is on clinical trials of WNA for the SP. WNA, or WNA combine with other conventional treatments were used as the intervention measures in the treatment group, while the control group was given conventional treatments such as drugs, placebo, sham acupuncture and no treatment, and so on.

#### 2.2.4 Types of outcome measures

##### 2.2.4.1 Primary outcome

The primary outcomes are effective rate, visual analog scale score.

##### 2.2.4.2 Secondary outcomes

(1) Constant-Murley score,
(2) Japanese Orthopaedic Association scores,
(3) Adverse events caused by WNA, such as scald, vomiting, weariness, dizziness, nausea, so on.

### 2.3 Search strategy

We will search for Pubmed, the Cochrane Library, Embase, CNKI, Wanfang Database, CBM and VIP Database from its inception to September 2021 with a language restriction on Chinese or English. The search adopts a combination of subject terms and free words, and the search strategy is determined after multiple pre-searches. We search the retrieval type are “warm needle acupuncture” or “needle warming moxibustion” and “Scapulohumeral periarthritis” Table 1 summarizes Pubmed preliminary search strategy, which will be adjusted according to the grammatical requirements of other electronic databases.

**Table 1:** Search strategy used in PubMed database.

Number	Search terms
#1	“scapulohumeral periarthritis”[MeSH Terms] OR “frozen shoulder”[MeSH Terms] OR “adhesive periarthritis of shoulder”[MeSH Terms] OR “periarthritis of shoulder”[MeSH Terms]
#2	“scapulohumeral periarthritis”[Title/Abstract] OR “frozen shoulder ”[Title/Abstract] OR “adhesive periarthritis of shoulder”[Title/Abstract] OR “periarthritis of shoulder”[Title/Abstract] OR “shoulder pain”[Title/Abstract] OR “Shoulder osteodystrophy”[Title/Abstract] OR “Shoulder osteoarthritis”[Title/Abstract] OR “Shoulder Periarthritis”[Title/Abstract] OR “Shoulder Pains”[Title/Abstract]
#3	#1 OR #2
#4	“warm needle acupuncture”[MeSH Terms] OR “warming acupuncture”[MeSH Terms] OR “needle warming moxibustion”[MeSH Terms]
#5	“warm needle acupuncture”[Title/Abstract] OR “warming acupuncture”[Title/Abstract] OR “needle warming moxibustion”[Title/Abstract] OR “needle warming moxibustion”[Title/Abstract] OR “needle warming”[Title/Abstract]
#6	#4 OR #5
#7	(“randomized controlled trial”[Publication Type] OR “controlled clinical trial”[Publication Type] OR “randomized”[Title/Abstract] OR “placebo”[Title/Abstract] OR “clinical trials as topic”[MeSH Terms] OR “randomly”[Title/Abstract] OR “trial”[Title]) NOT (“animals”[MeSH Terms] NOT “humans”[MeSH Terms])
#8	#3 AND #6 AND #7

This search strategy will be modified as required for other electronic databases.

### 2.4 Identification of studies

**Figure 1:**
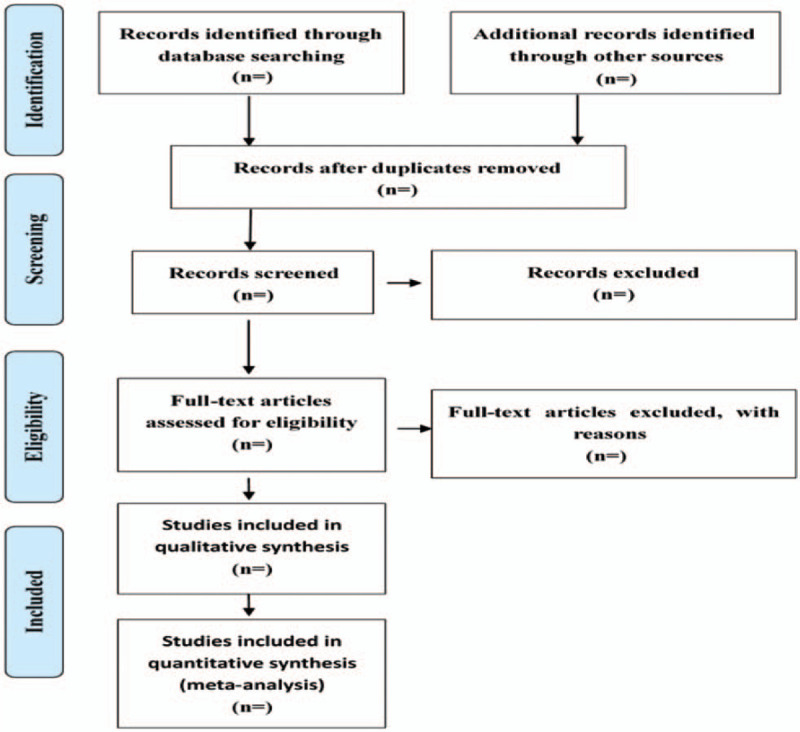
Flow diagram of studies identified.

Endnote X9 will be used for study selection. Two evaluators (WXY and HXH) conducted independent literature screening and cross-cutting. First, duplicate literature was excluded. For duplicate literature, the most comprehensive literature was selected. Then, the title and abstract were read to exclude obviously irrelevant literature. Finally, the full text was read to determine the final systematic review. To ensure the consistency of the literature screening, please cross-check the screening results and resolve any differences through discussion or negotiation with the third evaluator (JDL and YLJ). The details of the selection process are shown in Figure 1

### 2.5 Data collection

The two evaluators (WXY and HXH) conducted independent data extraction based on a predetermined data extraction table, and then cross-discussed to resolve any differences, or negotiated with the third evaluator (WQ and XGQ) to decide whether there was a difference. The following information and data were extracted from each included clinical trial: authors, time of publication, randomization method, blinding, number of cases of observation, diagnostic criteria, interventions and controls, duration of treatment, outcome measures (effective rate, visual analog scale score, Constant-Murley score, Japanese Orthopaedic Association scores, adverse events), and follow-up.

### 2.6 Risk of bias assessment

The quality of the included literature was assessed by 2 evaluators (WXY and LHN) using the risk of bias assessment tool recommended in the Cochrane Handbook 5.3,^[27]^ and if disagreements arose they were resolved by discussion or a third evaluator (LF and WQ) was consulted for a decision. Criteria included: correct use of randomisation; correct use of allocation concealment; correct use of blinding of patients; correct use of blinding of researchers; completeness of results and data; selective reporting of results; and presence of relevant bias. The standard for assessing the risk of bias adopts 3 levels of “low risk of bias,” “high risk of bias,” and “unclear risk of bias.”^[28]^

### 2.7 Statistical analyses

Meta-analysis was performed using RevMan 5.3 software. Relative risk and 95% confidence interval were used for the count data, standardized mean difference and 95% confidence interval were used for the measurement data as effect measures; if there was heterogeneity among the intervention protocols included in the studies, the heterogeneity test was performed between the studies using the karyotype test (test for α = 0.05), when *P* < 0.1 or *I*^2^ > 50%. perceived heterogeneity, using random-effects should model to calculate the risk ratio of the overall result; conversely, the fixed-effects model was used to calculate, and publication bias was analyzed using funnel plots. Subgroup analyses were performed based on factors that could be heterogeneous, and sensitivity analyses were performed when heterogeneity originated from low-quality studies.^[29]^

### 2.8 Analysis of subgroups or subsets

If the included studies are highly heterogeneous, we will perform a subgroup analysis based on age, sample size, methodological quality, and so on.

### 2.9 Sensitivity analysis

If heterogeneity is significant, we will conduct a sensitivity analysis to assess the robustness and quality of the findings by excluding each included study individually and varying the study's impact scale.

### 2.10 Ethics and dissemination

As the relevant data we extracted does not involve any personal privacy, we will not apply for ethical approval. We will publish this study to assess the validity and safety of WNA for SP and present it in a peer-reviewed journal or at a conference.

### 2.11 Summary of evidence

We will evaluate the quality of the evidence and classify it on 4 levels according to the Grades of Recommendations Assessment, Development and Evaluation (GRADE) guidelines: high, moderate, low or very low.^[30]^

## 3 Discussion

SP is a condition of limited shoulder movement, the severity of which can range from mild pain and/or mild limitation of movement to severe pain and/or severe limitation of movement, characterized by gradual onset of shoulder stiffness, severe pain (especially at night) and limited active and passive range of motion, and the main intervention is currently conservative treatment. Acupuncture is increasingly accepted by clinicians and patients in the treatment of musculoskeletal pain.^[31]^ In recent years, acupuncture and moxibustion have been widely used as a safe and non-obvious treatment for SP. Studies have shown that WNA can regulate the overall condition of the human body and is beneficial to analgesia.^[32]^ Besides, WNA is very safe to operate on heating and stimulating acupoints, with almost no side effects. This study conducted a systematic review and meta-analysis of the effectiveness and safety of WNA for the SP, and provided references for WNA treatment of SP. Besides, the agreement will also provide WNA clinical outcome indicators, treatment effects, adverse reactions and side effects.

The protocol has been developed under the PRISMA-P and is registered with the International Platform of Registered Systematic Review and Meta-analysis Protocols (INPLASY). It will follow the guidelines of the Cochrane Handbook of Systematic Intervention Reviews and the PRISMA-P statement.

## Author contributions

**Data curation:** Xiaoyu Wang, Xinghua Hai, Dongli Jiang, Lianjun Yin, Qi Wang, Guoqiang Xu.

**Formal analysis:** Xiaoyu Wang, Dongli Jiang, Lianjun Yin, Huanan Li.

**Funding acquisition:** Xinghua Hai, Qing Sun.

**Methodology:** Xiaoyu Wang, Huanan Li, Fang Liu.

**Project administration:** Xinghua Hai, Qing Sun.

**Validation:** Xiaoyu Wang, Dongli Jiang, Lianjun Yin.

**Writing – original draft:** Xiaoyu Wang, Dongli Jiang, Lianjun Yin.

**Writing – review & editing:** Xiaoyu Wang, Xinghua Hai, Huanan Li, Qing Sun.

## References

[1] Lundberg BJ The Frozen shoulder: clinical and radiographical observations the effect of manipulation under general anesthesia structure and glycosaminoglycan content of the joint capsule local bone metabolism. Acta Orthopaedica Scandinavica 1969;40 (sup119):1–59. 4952729

[2] Jones S Hanchard N Hamilton S A qualitative study of patients’ perceptions and priorities when living with primary frozen shoulder. BMJ open 2013;3 (9.):10.1136/bmjopen-2013-003452 PMC3787409 24078753

[3] Wu Z Yu X Xiong J Acupuncture and moxibustion therapy for scapulohumeral periarthritis: protocol for an overview of systematic reviews and meta-analysis. Medicine 2020;99 (35):10.1097/MD.0000000000021567 PMC7458254 32871872

[4] Dawson J Shepperd S Carr A An overview of factors relevant to undertaking research and reviews on the effectiveness of treatment for frozen shoulder. Shoulder & Elbow 2010;2:232–7.

[5] Huang C Xie L Lin Y Effectiveness and safety of fire needle on periarthritis of shoulder: Protocol for a systematic review and meta-analysis. Medicine 2019;98 (20.):10.1097/MD.0000000000015673 PMC6531137 31096502

[6] Zhang J Yuan W Chen C Different acupuncture therapies for treating periarthritis of the shoulder: overview of systematic reviews and network Meta-analysis. Chinese Tissue Engineering Res 2020;24:5723–32.

[7] Guo Y Guo J Progress in clinical rehabilitation treatment of frozen shoulder. Medical Rev 2014;20:2752–4.

[8] Juel NG Brox JI Brunborg C Very high prevalence of frozen shoulder in patients with type 1 diabetes of≥ 45 years’ duration: the Dialong Shoulder Study[J]. Archives of physical medicine and rehabilitation 2017;98:1551–9. 28219686 10.1016/j.apmr.2017.01.020

[9] Luime JJ Koes BW Hendriksen IJM Prevalence and incidence of shoulder pain in the general population; a systematic review[J]. Scandinavian J Rheumatol 2004;33:73–81. 10.1080/03009740310004667 15163107

[10] Huang CC Tsao SL Cheng CY Treating frozen shoulder with ultrasound-guided pulsed mode radiofrequency lesioning of the suprascapular nerve: two cases. Pain Med 2010;11:1837–40. 21040432 10.1111/j.1526-4637.2010.00970.x

[11] Jie W Fang X Zhang A Buccal acupuncture plus exercise therapy for scapulohumeral periarthritis[J]. Journal of Acupuncture and Tuina Science 2016;14:131–4.

[12] Wolf JM Green A Influence of comorbidity on self-assessment instrument scores of patients with idiopathic adhesive capsulitis. JBJS 2002;84:1167–73. 12107317

[13] Favejee MM Huisstede BMA Koes BW Frozen shoulder: the effectiveness of conservative and surgical interventions—systematic review[J]. British journal of sports medicine 2011;45:49–56. 20647296 10.1136/bjsm.2010.071431

[14] Grillet B Dequeker J Intra-articular steroid injection. Drug safety 1990;5:205–11. 2190596 10.2165/00002018-199005030-00005

[15] Godwin M Dawes M Intra-articular steroid injections for painful knees. Systematic review with meta-analysis[J]. Canadian Family Physician 2004;50:241–8. 15000335 PMC2214544

[16] Pareek A Chandurkar N Ambade R Efficacy and safety of etodolac-paracetamol fixed dose combination in patients with knee osteoarthritis flare-up: a randomized, double-blind comparative evaluation[J]. The Clinical journal of pain 2010;26:561–6. 20639739 10.1097/AJP.0b013e3181e15bba

[17] Lorbach O Anagnostakos K Scherf C Nonoperative management of adhesive capsulitis of the shoulder: oral cortisone application versus intra-articular cortisone injections[J]. Journal of shoulder and elbow surgery 2010;19:172–9. 19800262 10.1016/j.jse.2009.06.013

[18] Luo D Jr Liu Y Jr Wu Y Jr Warm needle acupuncture in primary osteoporosis management: a systematic review and meta-analysis[J]. Acup Med 2018;36:215–21. 10.1136/acupmed-2016-011227 PMC6089200 29986901

[19] Li X Han Y Cui J Efficacy of warm needle moxibustion on lumbar disc herniation: a meta-analysis[J]. Journal of evidence-based complementary & alternative medicine 2016;21:311–9. 26378088 10.1177/2156587215605419

[20] Ju ZY Wang K Cui HS Acupuncture for neuropathic pain in adults[J]. Cochrane Database of Systematic Reviews 2017;(12.):10.1002/14651858.CD012057.pub2 PMC6486266 29197180

[21] Li T Wang S Zhang S Evaluation of clinical efficacy of silver-needle warm acupuncture in treating adults with acute low back pain due to lumbosacral disc herniation: study protocol for a randomized controlled trial[J]. Trials 2019;20:470. 31366405 10.1186/s13063-019-3566-2 PMC6668190

[22] Li H Ji Y Zhang X Network Meta-analysis on 5 Acupuncture Methods in Treatment of Rheumatoid Arthritis [J]. Chinese J Trad Me 2020;38:154–9.

[23] Yang L Tan JY Ma H Warm-needle moxibustion for spasticity after stroke: a systematic review of randomized controlled trials[J]. International journal of nursing studies 2018;82:129–38. 29631145 10.1016/j.ijnurstu.2018.03.013

[24] Chen LA Liu HT Huang C Effectiveness and safety of warm needle acupuncture on children with cerebral palsy: Protocol for a systematic review and meta-analysis. Medicine 2019;98 (13.):10.1097/MD.0000000000014959 PMC6456086 30921197

[25] Zhao H Nie W Sun Y Warm needling therapy and acupuncture at meridian-sinew sites based on the meridian-sinew theory: hemiplegic shoulder pai. Evidence-Based Complementary and Alternative Medicine 2015;2015:10.1155/2015/694973 PMC4606215 26495023

[26] Shamseer L Moher D Clarke M Preferred reporting items for systematic review and meta-analysis protocols (PRISMA-P) 2015: elaboration and explanation. BMJ (Clinical research ed) 2015;350:g7647. 10.1136/bmj.g7647 25555855

[27] State Administration of Traditional Chinese Medicine. Standards for Diagnosis and Efficacy of TCM Diseases and Syndromes[M]. 1994;Nanjing: Nanjing University Press, 6.

[28] Higgins J Green SE Cochrane handbook for systematic reviews of interventions version 5.1.0. the cochrane collaboration (Eds). N Schmied Arch Pharmacol 2011;5:S38.

[29] Higgins JP Thompson SG Deeks JJ Measuring inconsistency in meta-analyses. British Med J 2003;327:557–60. 10.1136/bmj.327.7414.557 PMC192859 12958120

[30] Deng T Wang Y Huang D Methods for formulating clinical practice guidelines: GRADE method theory. Chine J Evid Based Cardiovasc Med 2018;10:1441–5.

[31] Green S Buchbinder R Hetrick SE Acupuncture for shoulder pain. Cochrane Database Syst Rev 2005;(2.):10.1002/14651858.CD005319 PMC12130916 15846753

[32] Chen B Zhang J Wu Y Therapeutic effect observation on combined tuina with warm needling moxibustion for adhesive shoulder periarthritis. Journal of Acupuncture and Tuina Science 2012;10:383–7.

